# Comparison of Real-world Outcomes Between Patients Treated with Tapentadol ER or Oxycodone CR

**DOI:** 10.36469/9905

**Published:** 2015-07-15

**Authors:** Mike Durkin, Jacqueline Pesa, Jessica Lopatto, Rachel Halpern, Damon Van Voorhis, Stephanie Korrer

**Affiliations:** 1 Janssen Scientific Affairs, LLC, Titusville, New Jersey, USA; 2 Janssen Scientific Affairs, LLC, Superior, Colorado, USA; 3 Medtronic, Inc., Mounds View, Minnesota, USA; 4 Optum, Health Economics and Outcomes Research, Eden Prairie, Minnesota, USA

**Keywords:** oxycodone cr, tapentadol er, resource use, health care costs, chronic pain

## Abstract

**Background:** The objective of this study was to compare health care utilization and costs between matched cohorts of chronic pain patients treated with the opioids tapentadol extended release (ER) or oxycodone controlled release (CR).

**Methods:** This retrospective study used claims data from the Optum Research Database. Commercial and Medicare Advantage adult patients with ≥1 prescription fill for oxycodone CR or tapentadol ER between September 1, 2011 and September 30, 2012 were eligible. The date of the first observed oxycodone CR or tapentadol ER claim was the index date. Patients had continuous health plan enrollment for 6 months before and after the index date, ≥ 90 days supply of opioid therapy, and no index drug claims in the preindex period. Patients were propensity score matched in a 1:2 ratio (tapentadol ER : oxycodone CR).

**Results:** The attributes of the matched cohorts (1,120 tapentadol ER and 2,240 oxycodone CR patients) appeared similar. In the 6 month post-index period, lower proportions of the tapentadol ER cohort than the oxycodone CR cohort had ≥1 inpatient stay (14.6% versus 20.5%; p<0.001) and ≥1 emergency department visit (33.4% versus 37.5%; p=0.021). The tapentadol ER compared with the oxycodone CR cohort had higher mean pharmacy costs ($4,263 versus $3,694; p <0.001), lower mean inpatient costs ($3,625 versus $6,309; p<0.001), and lower mean total healthcare costs ($16,510 versus $19,330; p=0.004).

**Conclusions:** During follow-up, total mean healthcare costs were lower among tapentadol ER patients than oxycodone CR patients, and tapentadol ER patients were less likely to have an inpatient admission or emergency department visit.

## INTRODUCTION

Chronic pain is estimated to affect about 100 million Americans[1], and may be associated with a variety of conditions, including osteoarthritis, rheumatoid arthritis, osteoporosis, low back pain, diabetic peripheral neuropathy, and fibromyalgia.[2] Of patients who report experiencing pain, those who are 45 or older are more likely to experience a long duration of pain (lasting 3 months or more) than are younger patients.[3] Pain can affect a patient's ability to perform routine household tasks and maintain social relationships, and can have a negative impact on a patient's emotional and physical well-being.[4,5] Conditions involving pain have been associated with high health care service utilization and medical costs in the US and other countries.[6,7]

Managing chronic pain is challenging due to the need for long therapy duration, differences in how individual patients respond to a drug, and potential for adverse drug events.[8] Opioids are a mainstay of pharmaceutical treatment for management of pain.[9] Tapentadol extended release (ER) is a long-acting opioid that received FDA approval in August 2011 and is indicated for treatment of pain severe enough to require daily, constant treatment, and for neuropathic pain associated with diabetic peripheral neuropathy.[10] Tapentadol is the first pharmacologic agent that affects two pathways involved in pain: it acts as an agonist of the mu-opioid receptor and inhibits neuronal reuptake of norepinephrine.[11] In clinical studies, tapentadol ER was found to have similar efficacy in reduction of chronic low back pain or osteoarthritis pain compared with oxycodone controlled release (CR).[12,13] However, rates of treatment-emergent adverse events (TEAEs) and discontinuation due to TEAEs were higher among oxycodone CR users than among tapentadol ER users.[12,13] Higher rates of adverse events could result in higher health care costs among patients, due to costs related to management of the adverse events, or costs related to inadequate management of pain if treatment needs to be discontinued.[14,15,16]

Some economic modelling studies on the health care costs of tapentadol ER relative to oxycodone CR have been published. Merchant et al. investigated the economic impact of placing tapentadol ER on a hypothetical US health plan formulary, and found that replacing a portion of oxycodone CR use with tapentadol ER resulted in a projected annual budget savings.[17] In another study, Neil et al. estimated the costs from a payer perspective associated with tapentadol ER versus oxycodone CR as initial treatment for chronic non-cancer pain.[18] Under their assumptions, annual average per-patient costs were lower among tapentadol ER patients than oxycodone CR patients. Finally, Coluzzi et al. used a Markov model to compare tapentadol ER with oxycodone CR among patients with musculoskeletal pain, and found that tapentadol ER was associated with lower health care costs and greater quality-adjusted life years compared with oxycodone CR.[19] We note that these previous studies modeled costs among hypothetical populations of patients using evidence from clinical trials and other data sources. However, we are not aware of prior studies that have compared health care costs and resource use for patients receiving oxycodone CR or tapentadol ER in a real-world treatment setting. The objective of this study was to compare health care utilization and costs between matched cohorts of chronic pain patients treated with tapentadol ER or oxycodone CR in a managed care setting.

## METHODS

### Database and Patient Identification

This retrospective study used the Optum Research Database (ORD), a proprietary research database containing medical and pharmacy claims data with linked enrollment information. Both commercial and Medicare Advantage health plan members were eligible for inclusion in this study. Patients were required to have a pharmacy claim for branded tapentadol ER (Nucynta ER) or oxycodone CR (OxyContin CR) between September 2011 and September 2012 (defined as the identification period).

The service date for the first observed claim for tapentadol ER or oxycodone CR during the identification period was set as the index date, and the drug on the claim was set as the index drug. To be included in the final study sample, subjects were also required to be ≥18 years old during the year of the index date, and to have 6 months of continuous health plan enrollment before and after the index date (defined as the pre-index period and post-index period, respectively). Patients were excluded if they had claims for more than one index drug (tapentadol ER or oxycodone CR) on the index date, or had any claims for the index drug during the pre-index period. To examine treatment of chronic pain rather than short-term or acute pain, patients were required to have been dispensed ≥90 days supply of opioid therapy, including any short- or long-acting opioids, during the 12 months of observation from 6 months prior to the index date to 6 months after the index date.

### Cohort Matching

Patients were assigned to a study cohort based on their index drug. Due to the potential effect that opioid abuse could have on health care utilization and cost, patients in both cohorts were stratified by the presence of evidence of opioid abuse. Opioid abuse was defined during the pre-index period based on presence of medical or pharmacy claims (≥1 medical claim with International Classification of Diseases, Ninth Revision, Clinical Modification [ICD-9-CM] diagnosis code 304.00-304.02, 304.70-304.72, 305.50-305.52; or ≥1 medical claim with CPT code 4306F; or ≥1 pharmacy claim for Suboxone or Subutex). For patients in each stratum (i.e., evidence of abuse and no evidence of abuse) of the tapentadol ER cohort, matches were sought from the corresponding strata of the oxycodone CR cohort through the application of a propensity score matching methodology.[20] Propensity scores were estimated with an unconditional logistic regression that incorporated predictors of the dependent variable, assignment to the tapentadol ER versus oxycodone CR cohort. For each patient's set of covariates, the propensity score represented the fitted value of the probability of being in the tapentadol ER index drug cohort. For each tapentadol ER patient, the two oxycodone CR patients with the closest propensity scores within a caliper of 0.01 were selected. If two qualified oxycodone CR patients were not found for a given tapentadol ER patient, that patient was excluded. All unmatched oxycodone patients were also excluded.

The logistic regression model for propensity score matching incorporated the following covariates observed in the pre-index period: age; gender; US census region; insurance type; pre-index Quan-Charlson comorbidity index score; pre-index all-cause health care costs; presence of pain conditions during the pre-index period (back and neck pain, neuropathic pain, cancer, other musculoskeletal pain); overall count of pain conditions; ≥2 short-acting opioid fills during the pre-index period; ≥2 long-acting opioid fills during the pre-index period; ≥1 medical claim for non-opioid substance abuse during the pre-index period; ≥1 medical claim for alcohol abuse during the pre-index period; ≥1 medical claim for a mental health condition during the pre-index period; and ≥1 pharmacy claim for a benzodiazepine during the pre-index period.

### Demographic and Clinical Characteristics

Patient age was defined as of the index year, and gender and geographic region were captured from enrollment information. A Quan-Charlson comorbidity score was calculated based on the presence of diagnosis codes on medical claims in the pre-index period.[21,22] Chronic pain conditions (including low back pain, cancer-related pain, diabetic peripheral neuropathy, neuropathic pain, and other musculoskeletal pain such as back and neck pain, rheumatism, arthritis, osteoarthritis, and rheumatoid arthritis) were identified based on the presence of at least two claims with a diagnosis code associated with that condition at least 30 days apart during the pre-index period. Fills of short- or long-acting opioids and of benzodiazepines were determined from pharmacy claims during the pre-index period. Non-opioid substance abuse, alcohol abuse, and mental health disorders were determined from medical claims during the pre-index period.

### Health Care Utilization

Numbers and counts of ambulatory visits (office visits or outpatient facility visits), emergency department visits, and inpatient stays were measured during the post-index period using medical claims. Counts of index drug fills and overall numbers of claims for short-acting opioids were determined from pharmacy claims.

### Health Care Costs

All-cause health care costs were computed as the combined health plan- and patient-paid amounts. Costs were calculated for the following categories: office visit costs; outpatient facility visit costs; emergency department costs; inpatient costs; other medical costs; medical costs (sum of office, outpatient facility, emergency department, inpatient, and other medical costs); pharmacy costs (costs of all prescription drug fills); and total health care costs (sum of all categories). Costs for expenditures in 2011 and 2012 were adjusted using the annual medical care component of the Consumer Price Index (CPI) to reflect inflation between 2011 and 2012; costs on claims with dates of service in the first 3 months of 2013 were not adjusted.

### Analysis

Pre-index characteristics were compared between the tapentadol ER and oxycodone CR cohorts both statistically and qualitatively prior to matching and between the cohorts following matching. A standardized difference (defined as the difference in the mean values of a given attribute between the cohorts divided by the square root of the pooled variance) of less than +/- 10% was considered a successful match.[23] Histograms depicting the distribution of propensity scores were generated for the two pre-match cohorts and the two matched cohorts, and compared qualitatively. Post-index period utilization outcomes and cost outcomes were compared between the matched cohorts. Differences in categorical outcomes between the matched cohorts were tested with Rao-Scott chi-square statistics.[24] Differences in continuous outcomes were tested with generalized linear models with robust variance estimators. To account for non-normally distributed costs, additional testing was performed; bootstrapped p-values for the differences in mean post-index costs between the tapentadol ER and oxycodone CR cohorts were computed using 10,000 matched bootstrapped samples.[25] No adjustments were made for multiplicity.

## RESULTS

In total, 1,148 patients with tapentadol ER as the index drug and 11,511 patients with oxycodone CR as the index drug were initially identified from the Optum Research Database as eligible for the study. Standardized differences between the tapentadol ER and oxycodone CR study-eligible cohorts were greater than 10% for most characteristics, and significant differences were observed between cohorts for many clinical and demographic characteristics (Table 1). Following matching at a 1:2 ratio, the matched study cohorts comprised 1,120 tapentadol ER patients and 2,240 oxycodone CR patients. Ninety-eight percent (98%) of the 1,120 tapentadol ER patients were successfully matched to 19% of the study-eligible oxycodone CR patients. Standardized differences between the matched cohorts were less than 10% for all characteristics found to be associated with treatment selection and used in the propensity score model (Table 2).

No statistically significant differences ($p<0.05$) were observed for those characteristics between the matched cohorts. Also, the distributions of propensity scores between the matched study cohorts were found to be similar based on comparison of histograms (Figure 1).

**Table 1. Pre-index Characteristics (Pre-Match)**

| | Tapentadol ER (N=1,148) | | Oxycodone CR (N=11,511) | | Standardized Difference (%) | p-value |
|---|---|---|---|---|---|---|
| | **Mean** | **SD** | **Mean** | **SD** | | |
| Age (continuous) | 51.27 | 12.60 | 56.37 | 14.04 | -38.28 | <0.0001 |
| Pre-index comorbidity index score | 0.70 | 1.34 | 1.49 | 2.21 | -43.28 | <0.0001 |
| Pre-index all-cause health care costs ($) | 16,049 | 34,177 | 30,969 | 57,172 | -31.68 | <0.0001 |
| | **n** | **%** | **n** | **%** | | |
| Gender | | | | | | |
| Female | 703 | 61.24 | 6,188 | 53.76 | 15.17 | <0.0001 |
| Male | 445 | 38.76 | 5,323 | 46.24 | -15.17 | <0.0001 |
| US census region | | | | | | |
| Northeast | 88 | 7.67 | 1,236 | 10.74 | -10.64 | 0.0003 |
| Midwest | 166 | 14.46 | 3,345 | 29.06 | -35.94 | <0.0001 |
| South | 779 | 67.86 | 4,721 | 41.01 | 55.96 | <0.0001 |
| West | 115 | 10.02 | 2,208 | 19.18 | -26.17 | <0.0001 |
| Other | 0 | 0.00 | 1 | 0.01 | -1.32 | 0.3173 |
| Insurance type | | | | | | |
| Commercial | 904 | 78.75 | 7,010 | 60.90 | 39.63 | <0.0001 |
| Medicare advantage | 244 | 21.25 | 4,501 | 39.10 | -39.63 | <0.0001 |
| Pre-index back and neck pain | 727 | 63.33 | 5,574 | 48.42 | 30.35 | <0.0001 |
| Pre-index other musculoskeletal pain | 487 | 42.42 | 5,959 | 51.77 | -18.80 | <0.0001 |
| Pre-index neuropathic pain | 411 | 35.80 | 2,546 | 22.12 | 30.51 | <0.0001 |
| Pre-index cancer | 37 | 3.22 | 1,286 | 11.17 | -31.12 | <0.0001 |
| Count of pre-index pain conditions | | | | | | |
| Zero | 222 | 19.34 | 2,309 | 20.06 | -1.81 | 0.5603 |
| 1 | 323 | 28.14 | 3,295 | 28.62 | -1.08 | 0.7266 |
| 2 | 296 | 25.78 | 2,917 | 25.34 | 1.02 | 0.7423 |
| 3 | 183 | 15.94 | 1,748 | 15.19 | 2.08 | 0.4974 |
| ≥4 | 124 | 10.80 | 1,242 | 10.79 | 0.04 | 0.9903 |
| ≥2 pre-index short-acting opioid fills | 1,024 | 89.20 | 9,998 | 86.86 | 7.22 | 0.0158 |
| ≥2 pre-index long-acting opioid fills | 240 | 20.91 | 1,764 | 15.32 | 14.53 | <0.0001 |
| Pre-index opioid abuse | 58 | 5.05 | 560 | 4.86 | 0.86 | 0.7788 |
| Pre-index non-opioid substance abuse | 59 | 5.14 | 489 | 4.25 | 4.21 | 0.1892 |
| Pre-index alcohol abuse | 18 | 1.57 | 330 | 2.87 | -8.83 | 0.0011 |
| Pre-index mental health disorder | 491 | 42.77 | 4,952 | 43.02 | -0.50 | 0.8706 |
| ≥1 pre-index benzodiazepine fill | 417 | 36.32 | 3,098 | 26.91 | 20.34 | <0.0001 |

ER: extended release, CR: controlled release, SD: standard deviation

**Figure 1.** Propensity Score Distributions

(A) Pre-Match Cohorts. One oxycodone CR patient with a non-matchable geographic area was dropped.

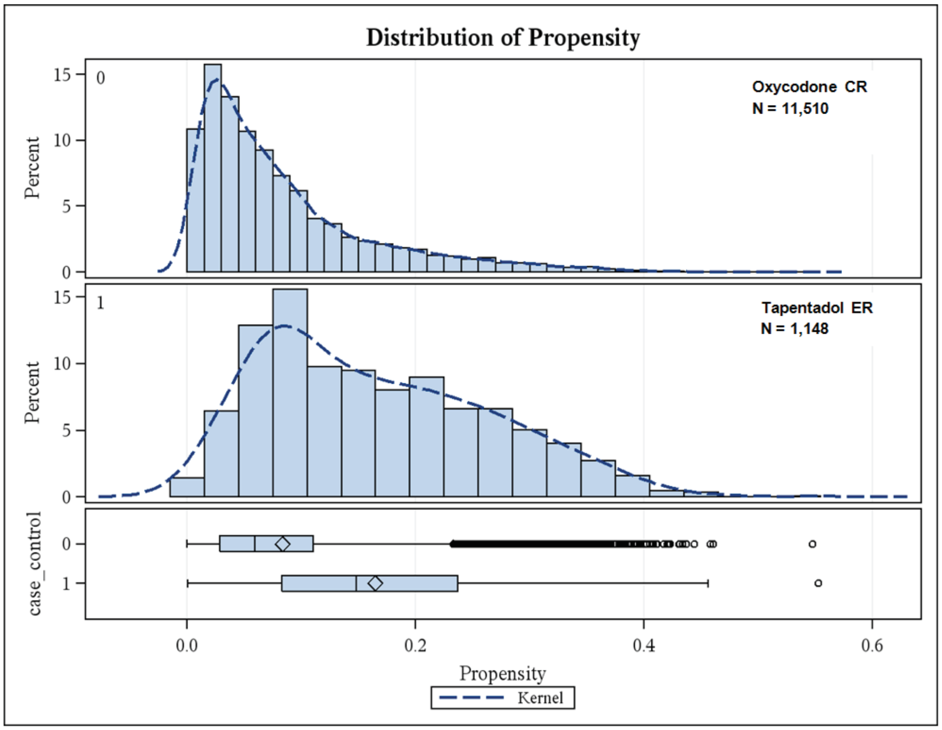

ER: extended release, CR: controlled release

(B) Post-Match Cohorts. All patients with at least one match are shown. Some tapentadol ER patients had only one oxycodone CR match and were excluded from the study population. Matching yielded 1,120 tapentadol ER patients and 2,240 oxycodone CR patients.

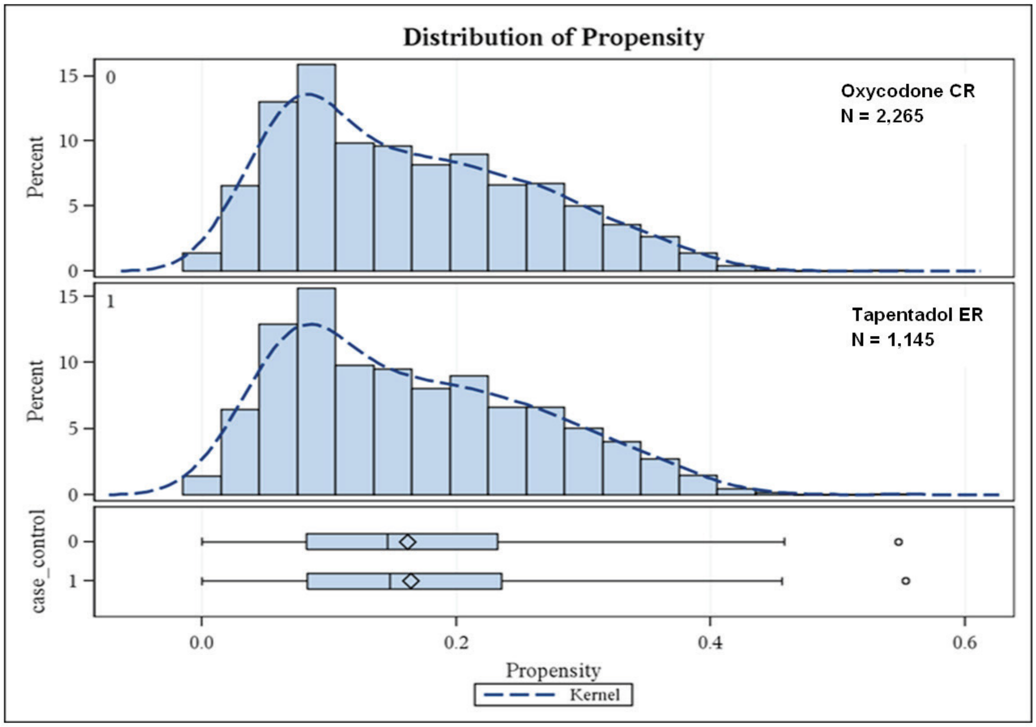

ER: extended release, CR: controlled release

**Table 2.** Pre-Index Characteristics (Post-Match)

| | Tapentadol ER (N=1,120) | | Oxycodone CR (N=2,240) | | Standardized Difference (%) | p-value |
|---|---|---|---|---|---|---|
| | **Mean** | **SD** | **Mean** | **SD** | | |
| Age (continuous) | 51.50 | 12.58 | 51.03 | 13.02 | 3.70 | 0.3144 |
| Pre-index comorbidity index score | 0.71 | 1.36 | 0.69 | 1.36 | 1.61 | 0.6604 |
| Pre-index all-cause health care costs ($) | 16,174 | 34,563 | 17,411 | 32,279 | -3.70 | 0.3178 |
| | **n** | **%** | **n** | **%** | | |
| Gender | | | | | | |
| Female | 678 | 60.54 | 1,367 | 61.03 | -1.01 | 0.7834 |
| Male | 442 | 39.46 | 873 | 38.97 | 1.01 | 0.7834 |
| US census region | | | | | | |
| Northeast | 88 | 7.86 | 183 | 8.17 | -1.15 | 0.7539 |
| Midwest | 166 | 14.82 | 287 | 12.81 | 5.82 | 0.1154 |
| South | 754 | 67.32 | 1,541 | 68.79 | -3.16 | 0.3871 |
| West | 112 | 10.00 | 229 | 10.22 | -0.74 | 0.8400 |
| Other | 0 | 0.00 | 0 | 0.00 | – | – |
| Insurance type | | | | | | |
| Commercial | 876 | 78.21 | 1,731 | 77.28 | 2.25 | 0.5391 |
| Medicare advantage | 244 | 21.79 | 509 | 22.72 | -2.25 | 0.5391 |
| Pre-index back and neck pain | 701 | 62.59 | 1,381 | 61.65 | 1.93 | 0.5979 |
| Pre-index other musculoskeletal pain | 478 | 42.68 | 974 | 43.48 | -1.62 | 0.6577 |
| Pre-index neuropathic pain | 388 | 34.64 | 767 | 34.24 | 0.85 | 0.8173 |
| Pre-index cancer | 36 | 3.21 | 70 | 3.13 | 0.51 | 0.8890 |
| Count of pre-index pain conditions | | | | | | |
| Zero | 222 | 19.82 | 469 | 20.94 | -2.77 | 0.4507 |
| 1 | 319 | 28.48 | 611 | 27.28 | 2.69 | 0.4618 |
| 2 | 281 | 25.09 | 548 | 24.46 | 1.45 | 0.6921 |
| 3 | 178 | 15.89 | 350 | 15.63 | 0.74 | 0.8407 |
| ≥4 | 120 | 10.71 | 262 | 11.70 | -3.11 | 0.3980 |
| ≥2 pre-index short-acting opioid fills | 998 | 89.11 | 1,983 | 88.53 | 1.84 | 0.6163 |
| ≥2 pre-index long-acting opioid fills | 232 | 20.71 | 477 | 21.29 | -1.42 | 0.6976 |
| Pre-index opioid abuse | 48 | 4.29 | 96 | 4.29 | 0.00 | 1.0000 |
| Pre-index non-opioid substance abuse | 57 | 5.09 | 109 | 4.87 | 1.03 | 0.7784 |
| Pre-index alcohol abuse | 18 | 1.61 | 34 | 1.52 | 0.72 | 0.8434 |
| Pre-index mental health disorder | 479 | 42.77 | 967 | 43.17 | -0.81 | 0.8246 |
| ≥1 pre-index benzodiazepine fill | 405 | 36.16 | 816 | 36.43 | -0.56 | 0.8791 |

ER: extended release, CR: controlled release, SD: standard deviation

In the matched cohorts, the average age of patients was 52 years for tapentadol ER cohort and 51 years for oxycodone CR. In both cohorts, the majority of patients were female (61%) and had commercial insurance (78% in tapentadol ER and 77% in oxycodone CR). In both the tapentadol ER and oxycodone CR cohorts, pre-index pain conditions included back and neck pain (63% and 62% of patients, respectively); other musculoskeletal pain (43% in both cohorts); neuropathic pain (35% and 34% respectively); and cancer (3%

in both cohorts). In both cohorts, 89% of patients had ≥ 2 pre-index short-acting opioid fills, and 21% had ≥2 pre-index long-acting opioid fills. Four percent of patients in each cohort had direct evidence of pre-index opioid abuse, and 5% in each cohort had evidence of pre-index non-opioid substance abuse. Mean pre-index all-cause health care costs were not significantly different between the matched tapentadol ER and oxycodone CR cohorts ($16,174 [$34,563] versus $17,411 [$32,279]). Post-index resource utilization was compared between the tapentadol ER cohort and the oxycodone CR cohort (Table 3).

**Table 3.** Post-index Health Care Utilization

| Post-index All-cause Utilization | | Tapentadol ER (N=1,120) | Oxycodone CR (N=2,240) | p-value |
|---|---|---|---|---|
| **≥ 1 Office visit** | n | 1,111 | 2,179 | |
| | % | 99.20 | 97.28 | <0.001 |
| **≥ 1 Outpatient facility visit** | n | 756 | 1,538 | |
| | % | 67.50 | 68.66 | 0.498 |
| **≥ 1 Emergency department visit** | n | 374 | 840 | |
| | % | 33.39 | 37.50 | 0.021 |
| **≥ 1 Inpatient stay** | n | 163 | 459 | |
| | % | 14.55 | 20.49 | <0.001 |
| **Count of office visits** | Mean | 12.68 | 13.88 | 0.002 |
| | SD | 10.26 | 11.85 | |
| | Median | 10.00 | 11.00 | |
| **Count of outpatient facility visits** | Mean | 5.04 | 5.52 | 0.103 |
| | SD | 7.78 | 8.55 | |
| | Median | 3.00 | 3.00 | |
| **Count of emergency department visits** | Mean | 0.94 | 0.95 | 0.911 |
| | SD | 2.63 | 2.23 | |
| | Median | 0.00 | 0.00 | |
| **Count of inpatient stays** | Mean | 0.19 | 0.31 | <0.001 |
| | SD | 0.56 | 0.78 | |
| | | 0.00 | 0.00 | |

ER: extended release, CR: controlled release, SD: standard deviation

The percentage of patients with an inpatient stay was significantly lower among the tapentadol ER cohort compared with the oxycodone CR cohort (14.55% versus 20.49%, p<0.001) and the average count of inpatient stays was also lower (0.19 versus 0.31, p<0.001). A lower percentage of patients in the tapentadol ER cohort had an emergency department visit relative to the oxycodone ER cohort (33.39% versus 37.50%, p=0.021). The mean number of index drug prescription claims was lower for the tapentadol ER cohort than for the oxycodone CR cohort (3.05 versus 3.60, p<0.001), as were the mean days supply of the index drug dispensed in the post-period (79.36 compared with 85.18, p=0.009). Lower proportions of the tapentadol ER cohort had at least one prescription (87.68% versus 94.73%, p<0.001) and at least two prescriptions (80.98% versus 90.63%, p<0.001) for a short-acting opioid compared with oxycodone CR cohort members (data not shown).

Mean health care costs during the post-index period were significantly lower among the tapentadol ER cohort compared with the oxycodone CR cohort ($16,510 versus $19,330, p=0.004) (Table 4). Mean medical costs were lower among the tapentadol ER cohort compared with the oxycodone CR cohort

($12,247 versus $15,636, p<0.001). Within medical costs the largest difference was seen in mean inpatient costs, which were significantly lower among the tapentadol ER cohort ($3,625 versus $6,309, p<0.001).

Mean pharmacy costs were higher among the tapentadol ER cohort compared with the oxycodone CR cohort ($4,263 versus $3,694, p<0.01).

**Table 4.** Post-index Health Care Costs

| Post-index All-cause Costs ($) | | Tapentadol ER (N=1,120) | Oxycodone CR (N=2,240) | p-value: t-test | p-value: matched bootstrap |
|---|---|---|---|---|---|
| **Office visit costs** | Mean | 2,212 | 2,720 | 0.010 | 0.012 |
| | SD | 3,992 | 7,622 | | |
| | Median | 1,291 | 1,241 | | |
| **Outpatient facility visit costs** | Mean | 4,753 | 4,677 | 0.878 | 0.879 |
| | SD | 14,115 | 12,831 | | |
| | Median | 556 | 702 | | |
| **Emergency visit costs** | Mean | 585 | 658 | 0.340 | 0.344 |
| | SD | 2,187 | 2,057 | | |
| | Median | 0 | 0 | | |
| **Inpatient costs** | Mean | 3,625 | 6,309 | <0.001 | <0.001 |
| | SD | 14,015 | 22,572 | | |
| | Median | 0 | 0 | | |
| **Other medical costs** | Mean | 1,073 | 1,271 | 0.155 | 0.164 |
| | SD | 3,454 | 5,111 | | |
| | Median | 208 | 267 | | |
| **Total medical costs** | Mean | 12,247 | 15,636 | <0.001 | <0.001 |
| | SD | 23,297 | 32,391 | | |
| | Median | 3,909 | 4,702 | | |
| **Pharmacy costs** | Mean | 4,263 | 3,694 | 0.001 | 0.002 |
| | SD | 5,147 | 4,410 | | |
| | Median | 3,027 | 2,482 | | |
| **Total health care costs** | Mean | 16,510 | 19,330 | 0.004 | 0.004 |
| | SD | 24,604 | 33,323 | | |
| | | 8,107 | 8,269 | | |

ER: extended release, CR: controlled release, SD: standard deviation

## DISCUSSION

When interpreting the findings of this study, limitations relating to claims data and the study design should be considered. Medical and prescription claims are generated for administrative purposes and may contain biases or inaccuracies that affect the study results. However, as we required 2 claims at least 30 days apart for identification of chronic pain conditions in this study, we expect in most cases that the chronic pain conditions were indicative of disease presence. The reliance on claims data does inhibit the generalizability of findings to health care resource use that is not submitted for reimbursement. It is important to distinguish the observed dispensing transaction from any actual medication-taking behavior for which we have no information.

This was not a randomized study. Therefore any real world selection bias that made it more likely for patients with certain characteristics to be prescribed one index medication rather than the other could also affect the outcomes observed. In order to address this concern, a propensity score matching methodology was applied to form two matched cohorts with similar pre-index characteristics. The effects of unobserved characteristics on selection bias and on the findings of this study remain unknown. For example, the severity, duration and frequency of pain cannot be observed through claims data. More information on patients' pain would not only aid in controlling for selection bias, but would offer an important outcome to examine as well. Without this information we can make no assessments of the clinical effectiveness of the study drugs. Chronic pain is not uniquely identifiable within a given insurance claim. To focus this analysis on chronic pain, the study was limited to patients dispensed ≥90 days supply of opioid therapy. The findings here cannot be generalized to chronic pain patients who are not receiving opioids. It is possible that additional clinical information could reveal that some study patients meeting this definition may not have chronic pain.

Significant differences were observed in the two study eligible cohorts prior to the matching process. Propensity score matching created tapentadol ER and oxycodone CR cohorts that were balanced on observed confounders of the relationship between opioid treatment and the outcomes, although the generalizability of results to broader populations of patients treated with oxycodone CR may be limited. In the absence of randomized, naturalistic trials, observational research with matched cohorts, such as this study, can support better informed population health decisions. The differences in economic outcomes observed between these two matched cohorts provide information that may be useful to US payers and health policy decision-makers.

The reasons for the between-cohort differences in the percentages of patients with all-cause ER and inpatient utilization and in mean inpatient, office visit, medical, pharmacy, and total costs can only be inferred. All-cause utilization and costs were selected as the outcome for this study because opioids can be used for a wide range of chronic conditions and many patients have comorbid chronic conditions; therefore, we were limited in our ability to identify meaningful condition-related utilization and costs. Differences in utilization and costs could be attributable to a range of factors including (but not limited to) rate of opioid-related adverse events, health status not measured with the propensity score covariates, or acute health care needs. Research is needed to better understand the factors associated with the utilization and cost differences we observed between the tapentadol ER and oxycodone CR cohorts. For example, research on the reasons for hospitalization and the types of inpatient services used would help illuminate reasons for the significant differences found in inpatient utilization and cost. Additional information on factors such as pain intensity, disease severity, and indicators of misuse would strengthen the analysis.

## CONCLUSIONS

Chronic pain patients in a large national managed care plan treated with tapentadol ER had lower total mean health care costs and were less likely to have a hospital admission or emergency department visit compared with a matched cohort of patients treated with oxycodone CR. Statistical controls were used to control for the effects of clinical, demographic and health-related factors available in the data, but the effects of other factors may still be significant. Further research is needed to understand the specific causality of hospital admissions and emergency department visits that account for much of the health care cost differences found in this study.

## ACKNOWLEDGEMENTS

Jesse Potash, Optum, provided medical writing assistance.

**Conflict of Interest Declaration**

This study was supported by Janssen Scientific Affairs, LLC. Michael Durkin and Jacqueline Pesa are employees of Janssen Scientific Affairs, LLC. Jessica Lopatto was a postgraduate fellow at Janssen Scientific Affairs, LLC at the time of this study. Rachel Halpern, Stephanie Korrer, and Damon Van Voorhis are employees of Optum. Optum contracted with and was paid by Janssen Scientific Affairs, LLC to conduct this study and write this manuscript.
